# A novel computational approach to isolating mechanisms of cognitive development across two systems: scene categorization and visually guided navigation

**DOI:** 10.1016/j.isci.2026.116570

**Published:** 2026-06-29

**Authors:** Zvi R. Shapiro, Alexander Weigard, Daniel D. Dilks

**Affiliations:** 1 Emory University, Psychiatry Department, Atlanta GA 30322, USA; 2 University of Michigan, Psychiatry Department, Ann Arbor, MI 48109, USA; 3 Emory University, Psychology Department, Atlanta GA 30322, USA

**Keywords:** linear ballistic accumulator, diffusion decision model, navigation, place recognition, drift rate

## Abstract

Computational models propose that cognitive development reflects greater evidence accumulation efficiency, but it remains unclear whether these gains stem from increased accumulation of correct information, reduced accumulation of incorrect information, or both—and if these processes differ across domains. Taking a computational approach, we tested these possibilities in two distinct cognitive systems: scene categorization and visually guided navigation. Ninety-five participants (ages 4–21) completed tasks in each domain. Accuracy and reaction times were fit with the linear ballistic accumulator model to estimate separate accumulation rates for correct and incorrect information. For scene categorization, only the correct accumulation rate increased with age, indicating a selective enhancement in accumulation efficiency. Conversely, for visually guided navigation, both correct and incorrect accumulation rates changed, reflecting broader efficiency gains. Together, these findings formally characterize the unique developmental mechanisms driving scene categorization and visually guided navigation while demonstrating the utility of this modeling approach across any cognitive system.

## Highlights

• Age gains in scene recognition reflects increased accumulation of correct information alone
• Visually guided navigation improves with age via more correct and less incorrect information
• This approach should be used to understand developmental change across cognitive systems

## Graphical abstract

**Figure:**
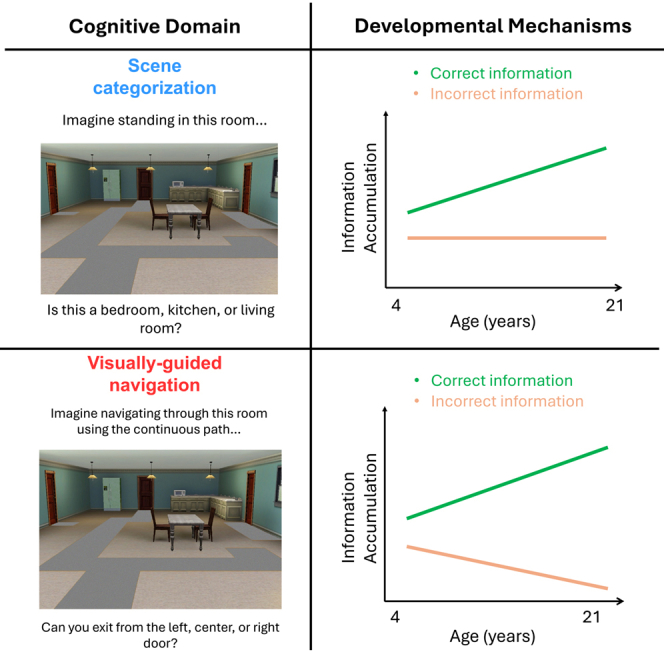

## Teaser

Behavioral neuroscience; Cognitive neuroscience; Computational bioinformatics; Developmental neuroscience; Psychology

## Introduction

Children’s cognitive abilities improve over development; across a wide variety of cognitive tasks, children’s accuracy rates increase with age while the latency and variability in their response times (RTs) decrease.^1^^,^^2^^,^^3^^,^^4^^,^^5^ But how? Studies addressing this question have leveraged evidence accumulation models—models that assume people gradually accumulate noisy evidence for each response option on a given task until reaching a threshold for one of the response options—most commonly the Diffusion Decision Model (DDM)^6^^,^^7^ (Figure S1, top). These studies consistently show that developmental improvement across diverse cognitive domains are largely attributable to increases in drift rate (*v*)—a single parameter indexing the average rate at which the evidence accumulation process moves toward the correct response boundary, and away from the incorrect response boundary.^8^^,^^9^^,^^10^^,^^11^^,^^12^^,^^13^

Another class of evidence accumulation models—race models^14^^,^^15^ have similar assumptions to the DDM—but instead explain performance as a result of the race between separate evidence accumulators for correct and incorrect responses until one reaches a decision threshold. The rate of evidence accumulation for the correct option is the correct drift rate (*vc*) and for the incorrect option is the error drift rate (*ve*) (Figure S1, bottom). Their difference then yields a single parameter—called the efficiency of evidence accumulation (EEA)^16^—which closely corresponds to the DDM drift rate.^17^ Importantly, EEA reflects two distinct components (1) the ability to accumulate correct (task-relevant) evidence and (2) the ability to avoid or reduce incorrect (task-irrelevant or misleading) evidence. Thus, developmental improvements in performance could stem from an increase in correct evidence accumulation, a decrease in incorrect evidence accumulation, or both. Clarifying these mechanisms could provide a more precise understanding of cognitive development. Here, we use a well-validated race model, the linear ballistic accumulator (LBA)^18^ to test these possibilities. Specifically, we modeled developmental change by estimating *vc* and *ve*. If children accumulate correct information more efficiently with age, *vc* should increase. Alternatively, if they become better at suppressing incorrect information, *ve* should decrease.

**Figure 1:**
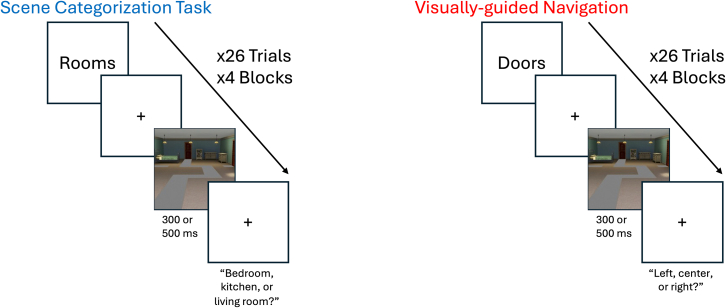
Workflow for the scene categorization and visually guided navigation tasks, as well as behavioral and computational outcomes Participants completed a total of 104 trials of each task. Mean accuracy and response times were then used to compute LBA parameters.

As test cases, we applied the LBA to two cognitive abilities (ages 4–21) with distinct neural substrates (Figure 1): scene categorization (i.e., our ability to recognize places)—supported by the parahippocampal place area (PPA)—and visually guided navigation (i.e., our ability to move about the immediately visible environment, avoiding boundaries and obstacles)—supported by the occipital place area (OPA).^19^^,^^20^^,^^21^^,^^22^^,^^23^^,^^24^^,^^25^^,^^26^ Critically, in addition to having distinct neural substates, these systems also develop along different timelines, with scene categorization developing earlier than visually guided navigation.^27^^,^^28^^,^^29^^,^^30^ Given these distinct developmental trajectories, it is therefore not unreasonable to expect different underlying developmental mechanisms for these systems. If such differences are indeed found, they would not only formally characterize the mechanisms underlying the development of scene categorization and visually guided navigation beginning at age 4, but also provide converging evidence to the model’s utility for isolating the mechanisms of development.

In addition, we also expected boundary (*b*) to decrease with age, consistent with prior DDM studies.^8^^,^^10^^,^^11^ Note, however, all else held equal, a decrease in boundary leads to a decrease in accuracy (not an increase as we are trying to explain here), though this reduction may be offset by increases in drift rate, allowing faster responses while maintaining or improving accuracy.^31^ Lastly, we also evaluated changes in nondecision time (*t*_*er*_), a parameter reflecting nondecision task components (stimulus encoding and motor RT), to account for the possibility that change in these processes may contribute to task performance.

## Results

### Behavioral summary measures

**Figure 2:**
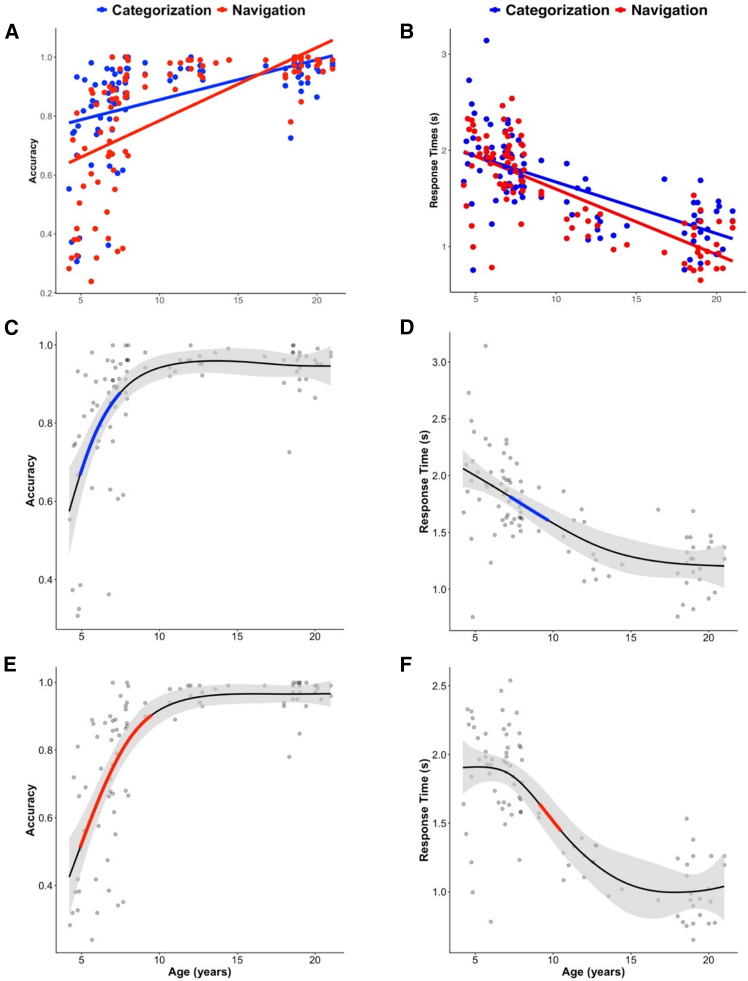
Age-related improvement in performance on the scene categorization and visually guided navigation tasks Changes in accuracy (A) and reaction time (B) over age for the scene categorization task (blue line) and the visually guided navigation task (red line). The same data points (split by task) are presented in (C–F) with generalized additive models showing change in accuracy and response times on the scene categorization (C and D) and the visually guided navigation (E and F) tasks, with red and blue segments indicating ages at which significant developmental change occurs (*p* < 0.05), respectively. Shaded gray segments reflect the 95% CI.

Before providing a formal account of how scene categorization and visually guided navigation develop, we first sought to confirm that we could replicate prior work^27^ showing these abilities develop along different timelines, as indicated by traditional behavioral summary measures. Indeed, a linear trend analysis on accuracy revealed a significant difference between the developmental trajectories for scene categorization and visually guided navigation (*F*(1,90) = 24.37, *p* < 0.001, ηp2 = 0.21; Figure 2A), with performance on the visually guided navigation task taking longer to develop (i.e., having a steeper slope) than on the scene categorization task. The same pattern remained for both the easy (*F*(1,90) = 22.85, *p* < 0.001, η_p_^2^ = 0.20) and hard (*F*(1,90) = 20.66, *p* < 0.001, η_p_^2^ = 0.19) conditions. We also found a similar pattern for RT, with RTs on the visually guided navigation task taking longer to develop than for the scene categorization task (*F*(1,90) = 7.03, *p* < 0.01, η_p_^2^ = 0.07; Figure 2B). Again, the same pattern remained for both the easy (*F*(1,90) = 8.46, *p* < 0.01, η_p_^2^ = 0.09) and hard conditions (*F*(1,73) = 4.80, *p* < 0.05, η_p_^2^ = 0.05). Having found the same pattern of results across the easy and hard conditions, we computed and utilized the mean parameter values for *vc* and *ve* across the difficulty conditions in subsequent analyses.

However, before proceeding, we need to rule out the possibility that our results were due to reduced left-right confusion on the visually guided navigation task with increasing age. While we do not believe that such confusion explain our findings since we exactly replicated a prior study that critically ruled out such left-right confusion (Kamps, Rennert et al.^27^). We nevertheless evaluated accuracy on each trial type (left, center, or right) within the visually guided navigation task in our youngest age group (children age 4; *n* = 11), who would be most likely to show such confusion. If left-right confusion was driving younger children’s reduced performance on the visually guided navigation task, we would expect performance on the center door to be better than performance on the left and right doors. We did not, however, find an effect of trial type (F(1.81, 16.33) = 0.98, *p* = 0.389, η^2^_*p*_ = 0.10). Next, we also evaluated whether children’s response pattern differed from our adult participants (ages 18+; *n* = 23). Crucially, however, we did not find such group by trial type interaction (F(2,62) = 1.99, *p* = 0.15, η2*p* = 0.06), suggesting that children exhibit no more left-right confusion on this task than adults.

Finally, having now replicated previous findings and confirmed our results were not due to simple left-right confusion, we then evaluated the precise developmental window for each task in terms of accuracy and RTs by estimating simultaneous confidence intervals (SCIs) for each on both tasks and identifying those ages in which the derivative was reliably different from zero (see STAR Methods). We found that significant change in accuracy for the scene categorization task was first evident at age 4.8 and continued until age 7.6 (Figure 2C), while changes in RT began at age 7.2 and continued until age 9.7 (Figure 2D). Note that accuracy preceding RT—as shown here—is consistent with previous research.^32^^,^^33^ For the visually guided navigation task, we found significant change in accuracy rates between ages 4.8–9.5 (Figure 2E), and in RTs between ages 9.1–10.5 (Figure 2F). Together, these results provide further evidence that these systems develop along different, dissociable timelines.

### Model description of behavior

**Figure 3:**
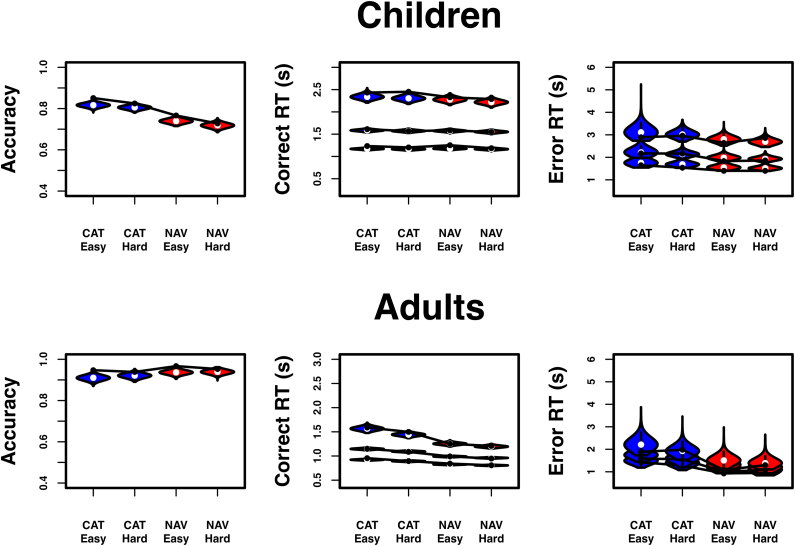
Posterior predictive plots comparing the predicted and empirical accuracy and RTs (correct and error) produced by the study participants Child data are presented in the top row and adult data in the bottom row with the accuracy and RTs (correct and error) generated by the model parameters for the scene categorization (CAT in blue) and visually guided navigation (NAV in red) tasks across easy and hard conditions. Black points and lines represent empirical data while violin density plots represent the range of predicted values at the 0.1, 0.5, and 0.9 quantiles. The density and spread of the violin plots represent uncertainty in predictions of the model due to uncertainty about the values of its parameter estimates. Concordance between model-predicted values and empirical data points indicates the model can account for task effects on behavior.

Before evaluating the LBA parameters, we ensured that our model fit our data, accounting for participants’ responding in each of our tasks. To that end, we compared the accuracy and RTs generated by our model parameters with the empirical accuracy and RTs produced by the study participants. To do so, we drew 1,000 samples from the posterior distribution of model parameters to predict participants’ accuracy and RTs (for correct and error responses). Posterior predictive plots^34^ comparing data generated from the model with empirical data for group average accuracy rates and group average correct and error RT quantiles (0.1, 0.5, and 0.9) in children and adults are displayed in Figure 3. These plots allow for model fit to be gauged in terms of absolute discrepancies between model-predicted values and empirical data points, the latter of which should be within the posterior predictive distributions predicted by the model (represented by the gray violin density plots), as well as in terms of whether the model is able to explain key directional trends in the data (e.g., experimental effects).

First, regarding absolute discrepancies, for both children and adults, the vast majority of empirical data points fell within the posterior predictive distributions. Broader density plots for error RTs represent greater uncertainty given the relatively low number of error RTs compared to correct RTs, but the location of the empirical data points within these predicted distributions indicates that the model still displays a reasonable account of these data. The handful of instances in which empirical data points fall outside of the posterior predicted distributions (e.g., some accuracy rates and correct RT quantiles) are values for which the model predictions have far less uncertainty, and therefore the absolute deviations are quite small in practical terms (e.g., the model underestimates accuracy in the easy condition of the scene categorization task by <5%).

Second, regarding the model’s explanation of directional trends in the child and adult data, the model provided an excellent account of the between-task accuracy and RT effects (greater accuracy and slower RTs during the scene categorization task compared to the visually guided navigation task). Taken together then, despite the small and few absolute deviations between model predictions and empirical data, the model provides a good account of the effects of task on the fine-grained details of participants’ behavior.

### LBA parameters

**Figure 4:**
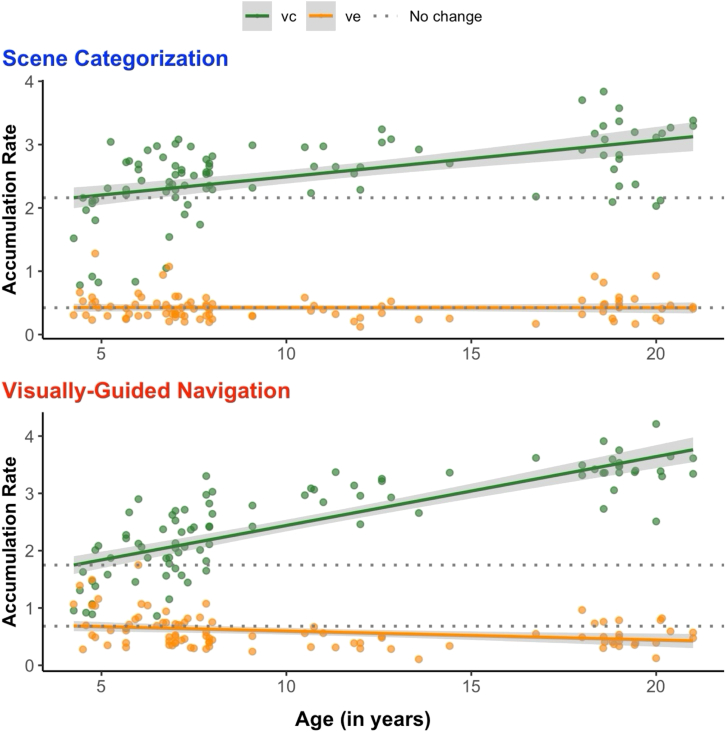
Age-related changes in *vc* and *ve* in the scene categorization and visually guided navigation tasks In the scene categorization task (top) *vc* (green line) changed with age (*p* < 0.001) but *ve* (orange line) did not (*p* = 0.96). In the visually guided navigation task (bottom), both *vc* (*p* < 0.001) and *ve* (*p* < 0.01) changed with age. Gray dotted lines represent no change (slope = 0). Shaded gray segments reflect the 95% CI.

Having now confirmed that our model adequately represents our empirical data, we turned to the LBA and asked whether an increase in correct information (*vc*) and/or a reduction in incorrect information (*ve*) is driving greater efficiency with age in each of our two model systems. Turning first to the scene categorization task, we examined whether age-related change is present in our primary parameters of interest: *vc* and *ve* (Figure 4, top). An increase in *vc* with age was found (*F*(1,90) = 34.49, *t*(90) = 5.873, *p* < 0.001, *R*^*2*^ = 0.28), consistent with the idea that cognitive development is driven by an increase in correct information. In contrast, when we evaluated *ve* in the scene categorization task, we found no association with age (*F*(1,90) = 0.003, *t*(90) = −0.055, *p* = 0.96, *R*^*2*^ = 0. 00003).

We next asked whether the changes we observed in each parameter took place within the developmental window of scene categorization. Indeed, the pointwise confidence interval (PCI) for *vc* at the median age (7.3 years old) of the developmental window for the scene categorization task was significantly increasing (99% confidence interval, CI = [0.02, 0.25]). No such increase was found at the median post-developmental age. By contrast, we found no change in the PCI for *ve* within either the developmental (age = 7.3, 99% CI = [−0.05, 0.02] or post-developmental window (age = 15.9, 99% CI = [−0.02, 0.04]). Taken together with the findings of our behavioral summary measures, the scene categorization system matures within a limited developmental window. Within this time frame, the reduction of incorrect information was already minimal and an increase in the accumulation of correct evidence uniquely contributed to improvement in task performance.

For the visually guided navigation task (Figure 4, bottom), we found age significantly predicted an increase in *vc* (*F*(1,90) = 165.90, *t*(90) *=* 12.88, *p* < 0.001, *R*^*2*^ = 0.65), reflecting an increase in correct information. Unlike scene categorization, we also found a decrease in *ve*, (*F*(1,90) = 8.43, *t*(90) *=* −2.90, *p* < 0.01, *R*^*2*^ = 0.09), reflecting a reduction in incorrect information. Evaluating the PCIs, we found evidence of a significant increase in *vc* (99% CI = [0.13, 0.37]) as well as a decrease in *ve* at the median age (age = 7.7, CI = [−0.14, −0.01]) within the visually guided navigation task’s developmental range. There was no reliable change in *vc* (CI = [−0.07, 0.15]) or *ve* (CI = [−0.03, 0.08]) when evaluated at the median (16.3 years old) of the post-developmental age range. Thus, development of visually guided navigation was associated with an increase in the accumulation of correct information as well as a reduction in information that is incorrect, each restricted to the developmental window for this task.

Next, as predicted, *b* (Figure S2) for the scene categorization task significantly decreased with age, (*F*(1,90) = 79.76, *t*(90) *= −*8.93, *p* < 0.001, *R*^*2*^ = 0.46). Likewise, *b* for the visually guided navigation task significantly decreased with age, *F*(1,90) = 98.69, *t*(90) *= −*9.93, *p* < 0.001, *R*^*2*^ = 0.52). These results show that participants required less evidence before responding on either task.

Finally, there was no change in *t*_*er*_ (Figure S3) with age on the scene categorization task (*F*(1,90) = 0.004, *t*(90) = 0.06, *p* = 0.95) nor on the visually guided navigation task (*F*(1,90) = 0.05, *t*(90) = 0.23, *p* = 0.82). Thus, nondecision components, such as stimuli encoding efficiency, did not appear to change with age.

## Discussion

Using an evidence accumulation model in a novel way, we investigated two computationally derived mechanisms that could explain developmental improvements. Specifically, we asked whether such improvements were driven by an increased accumulation of correct information, reduced accumulation of incorrect information, or both in two cognitive systems—scene categorization and visually guided navigation. As scene categorization and visually guided navigation have been differentiated both in the brain and in their developmental trajectories, we hypothesized their development may rely on different mechanisms. Indeed, while the development of scene categorization across ages 4–21 was driven by an increase in the accumulation of correct information alone, the development of visually guided navigation was associated with both an increase in the accumulation of correct information and a decrease in error information. Note, however, it is still possible that the development of scene categorization is accompanied by a reduction in the accumulation of incorrect information but was not observed because such a reduction may have occurred at a younger age than that sampled in this study. If true, this possibility raises the intriguing hypothesis that the development of any cognitive system may be explained by a rapid reduction in the accumulation of incorrect information along with a protracted increase in the accumulation of correct information.

What do these changes reflect cognitively? EEA has been proposed as a trait-like ability,^16^^,^^35^^,^^36^ reflecting a signal-to-noise ratio^37^ or the balance between task-relevant and task-irrelevant information.^17^ Within this framework, developmental changes in EEA during ages 4–21 can be interpreted as shifts in this balance: scene categorization appears to develop through the strengthening of signal/task-relevant evidence alone, whereas visually guided navigation develops through both the enhancement of signal/task-relevant evidence and the suppression of task-irrelevant information.

How might these developing mechanisms be instantiated neurally? Although the neural basis for these changes is unknown, a straightforward interpretation might be that, in the age range examined here, within both PPA (supporting scene categorization) and OPA (supporting visually guided navigation) increases in the accumulation of correct information is due, for example, to further myelination or neuronal tuning. By contrast, the selective decrease in accumulation of incorrect information in visually guided navigation may result from pruning or increased inhibition of spontaneous neuronal firing in OPA as the region matures.

Recent work has also suggested the origin of evidence in this framework could reflect the output of stochastic convolutional neural networks (CNNs) that have been trained to classify stimuli on the basis of relevant visual features.^38^ Hence, when using visual information about scene categories or visually guided navigation to guide responses, neurons within PPA and OPA, respectively, could act as specialized classifiers that generate evidence in favor of classifying a visual stimulus within the range of categories available to that region (e.g., “kitchen” or “path on the right”). This view would suggest that the classification process involves increasing identification of relevant features for both scene categorization and visually guided navigation, but differs in reducing identification of incorrect information for visually guided navigation alone.

In conclusion, this study is, to our knowledge, the first study to use a computational model, here the LBA, to identify the precise mechanisms underlying age-related improvement of two different cognitive systems. The same approach can be applied to determine the factors driving developmental change in a variety of domains, and we call for future work to do so.

### Limitations of the study

While the LBA provides an intentionally parsimonious description of the underlying decision process, this level of abstraction was necessary to successfully dissociate developmental changes in the accumulation of correct and incorrect information. Because the mechanisms identified here are currently constrained to computational definitions, future work must integrate these models with neural measures. Doing so will clarify how developmental shifts in evidence accumulation correspond to the physical maturation of cortical networks involved in scene categorization and visually guided navigation. Furthermore, extending this modeling approach to additional cognitive domains will be critical for determining whether these developmental trajectories rely on domain-general or domain-specific computational mechanisms.

## Resource availability

### Lead contact

Requests for further information and resources should be directed to and will be fulfilled by the lead contact, Daniel D. Dilks (dilks@emory.edu).

### Materials availability

This study did not generate new unique materials.

### Data and code availability

• Participant data have been deposited at Open Science Framework (OSF) and will be publicly available as of the date of publication. OSF: https://doi.org/10.17605/OSF.IO/JMV7B.
• All original code has been deposited at Open Science Framework (OSF) and will be publicly available as of the date of publication. OSF: https://doi.org/10.17605/OSF.IO/JMV7B.
• Any additional information required to reanalyze the data reported in this paper is available from the lead contact upon request.

## Acknowledgments

This work was supported by the 10.13039/100000053National Eye Institute grant number EY29724 (D.D.D.) and 10.13039/100000026National Institute on Drug Abuse grant number K23 DA051561 (A.W.).

## Author contributions

Conceptualization and methodology, Z.R.S. and D.D.D..; investigation, formal analysis, data curation, visualization, and writing – original draft. Z.R.S.; writing – review and editing, A.W. and D.D.D.

## Declaration of interests

The authors declare no competing interests.

## STAR★Methods

### Key resources table

**Table:** 

REAGENT or RESOURCE	SOURCE	IDENTIFIER
**Deposited data**		

Participant data	Open Science Framework	https://doi.org/10.17605/OSF.IO/JMV7B

### Experimental model and study participant details

#### Participants

Seventy-two children (40% female) between the ages of four and 16 (mean age = 7 years 8 months; range = 4 years 3 months–16 years 8 months) were recruited from the urban and suburban counties surrounding Emory University. One child refused to complete the tasks after providing assent, and data for two additional children was not recorded due to computer error. Five children completed only one block (25%) of the task. Analyses below are reported with these five children as the pattern of results did not differ when they were excluded. In addition, we recruited 23 undergraduate Emory University students (78% female; mean age = 21 years; range = 19–21 years) who received course credit for their participation, leaving a total of ninety-two participants with complete data.

#### Ethics statement

This study was approved by Emory University (IRB #00069523). Informed consent was obtained all participants over the age of 18. Verbal or written assent was obtained from all children ages 4–17, along with parent consent for study participation.

### Method details

#### Procedure

Testing was conducted at Emory University in Atlanta, GA. Participants completed both a scene categorization task and a visually-guided navigation task (Figure 1), as used in an adult fMRI study (21) and a developmental behavioral study (27). Briefly, to make the tasks more understandable and accessible to children, the scene categorization task was referred to as the “rooms” game, while the visually-guided navigation task was referred to as the “doors” game. During the “rooms” game, participants were asked to imagine standing in the room and had to indicate what kind of room they were standing in (i.e., a “bedroom,” “kitchen,” or “living room”). During the “doors” game, participants were asked to imagine that they were walking on a continuous path through the room and had to indicate whether they could leave through the door on the left, center, or right wall.

The two tasks were matched on difficulty in typically developing adults (21), and in all other aspects of the design, stimuli, and procedure. Participants completed a short training phase of practice trials for each task immediately before the testing phase, as reported in (27). For the testing phase, both tasks were performed on the same set of 36 images, which were identical to those used in (21) and (27).

Participants completed 4 blocks of each task (8 blocks in total), and the order of tasks was counterbalanced across participants. An instruction screen appeared at the start of each block to remind children of the game/task (i.e., rooms/scene categorization or doors/visually-guided navigation). Each block consisted of 26 experimental trials, and each trial consisted of a stimulus presentation, followed by a fixation screen, during which the participant gave a nonspeeded three-alternative forced choice response. Within each task, difficulty was varied to ensure sufficient error rates across participants and facilitate parameter recovery, by presenting half the images for 500 ms (“easy”) and the remaining images for 300 ms (“hard”). Difficulty was randomized within each condition, such that the same images were presented in each difficulty condition but in random order. The next trial began 500 ms after the participant had responded. For both tasks, participant responses and response times were recorded via button press. More specifically, participants responded by pressing one of three buttons on a Cedrus RB-540 response box. In the scene categorization task, the three response buttons associated with each door were laid in an inverted “V” shape, with a different button corresponding to each response option (bedroom, kitchen, and living room). Additionally, each button was labeled with a picture of an object relevant to that room (a bed, a stove, and a sofa). Participants used the same button layout to respond during the visually-guided navigation task, which aligned with the relative spatial location of each door on the screen and participants were encouraged to match their response on the keypad with the direction of the correct door on the screen (mimicking a pointing response). In this task, each button was labeled with a picture of a door to help participants remain on task.

### Quantification and statistical analysis

We first evaluated the developmental trajectories of accuracy and RTs (linear trend analysis) using an analysis of covariance with task as a within-subject factor and age as a covariate to replicate previous findings showing performance on these tasks exhibit different developmental trajectories. Second, we then determined the precise age range (“developmental window”) where these abilities show significant change. Following other work examining developmental windows (39–41), we fit a generalized additive model (GAM) (42) to accuracy rates and RTs in each task and obtained 10,000 simulations of the model coefficients, from which we constructed simultaneous 95% confidence intervals (SCI) – accounting for multiple comparisons – for all time points at a 0.1 year resolution which allowed us to identify the age range at which the derivatives were reliably above zero thus reflecting the estimated age range at which significant development occurs (43, 44).

Next, LBA parameter estimates were obtained using participants’ accuracy and RT (recorded from the onset of each image) on individual trials. Stimuli presentation times (300 and 500 ms) on these tasks were relatively brief, such the majority of the evidence accumulation process occurred after the images had disappeared. Very short (<300 ms) and long (>5500 ms) RTs (2.98% of scene categorization task trials 4.02% of children’s trials and 0.13% of adult trials for the scene categorization task, and1.97% of visually-guided navigation trials 2.65% of children’s trials and 0.09% of adult trials for the visually-guided navigation trials) were excluded to remove fast guesses and trials in which participants were not engaged in the task. Of particular interest in the current study were the correct evidence accumulator (vc), error evidence accumulator (ve), and boundary (b), which were separately estimated for each participant from each task. Modeling was implemented using Dynamic Models of Choice (45), a free set of R functions for conducting model-based choice RT analyses in a Bayesian framework. A more detailed account of the modeling procedure along with LBA parameters (Table S1) is provided in the supplemental materials. Briefly, our model assumes an evidence accumulator and a boundary for each response option, such that each trial consisted of three accumulators racing toward three boundaries. We assumed the accumulator for the correct response was drawn from a distribution with mean = vc and SD = svc, and the accumulators for the two incorrect responses for each trial were both drawn from a distribution with mean = ve and SD = sve.Briefly, in each task we obtained separate evidence accumulators for correct and incorrect responses as well as response boundaries. We additionally conducted a recovery study to confirm that our model is capable of accurately estimating parameters given the number of trials and design features of our experimental task (46). For both tasks, parameter values showed acceptable to good recovery with no apparent bias (Figure S2). Further details are provided in the supplemental materials.

Finally, we were unable to estimate a reliable developmental window for the LBA parameters due to the greater inherent uncertainty in their estimation than in directly observed accuracy and RT data. Therefore, we first regressed each LBA parameter on age to determine whether any significant change was evident. And second, to confirm that changes in our parameters fell within the developmental windows that we observed in accuracy and RT, we evaluated the 99% pointwise confidence interval (PCI) for the derivatives of each parameter at the median age of the developmental window for each task, relative to a “post-developmental window.” This post developmental window was defined as the range between the oldest age in which we observe change in our behavioral measures and end of our age range.
