# Supplementary material for: A novel computational approach to isolating mechanisms of cognitive development across two systems: scene categorization and visually guided navigation
Source: iScience. 2026 Jun 29;29(7):116570. doi: 10.1016/j.isci.2026.116570 (PMC13378356; doi:10.1016/j.isci.2026.116570)
Supplement: Document S1. Figures S1–S3, Table S1 and Data S1 [file mmc1.pdf]

iScience, Volume *29*

Supplemental information

A novel computational approach to isolating mechanisms of cognitive development across two systems: scene categorization and visually guided navigation

Zvi R. Shapiro, Alexander Weigard, and Daniel D. Dilks

Fig. S1. Age-related changes in *b* (boundary). The top panel depicts change in *b* over age in the scene categorization task and the bottom panel depicts change in *b* in the visually-guided navigation task. Related to Figure 4 and STAR Methods.

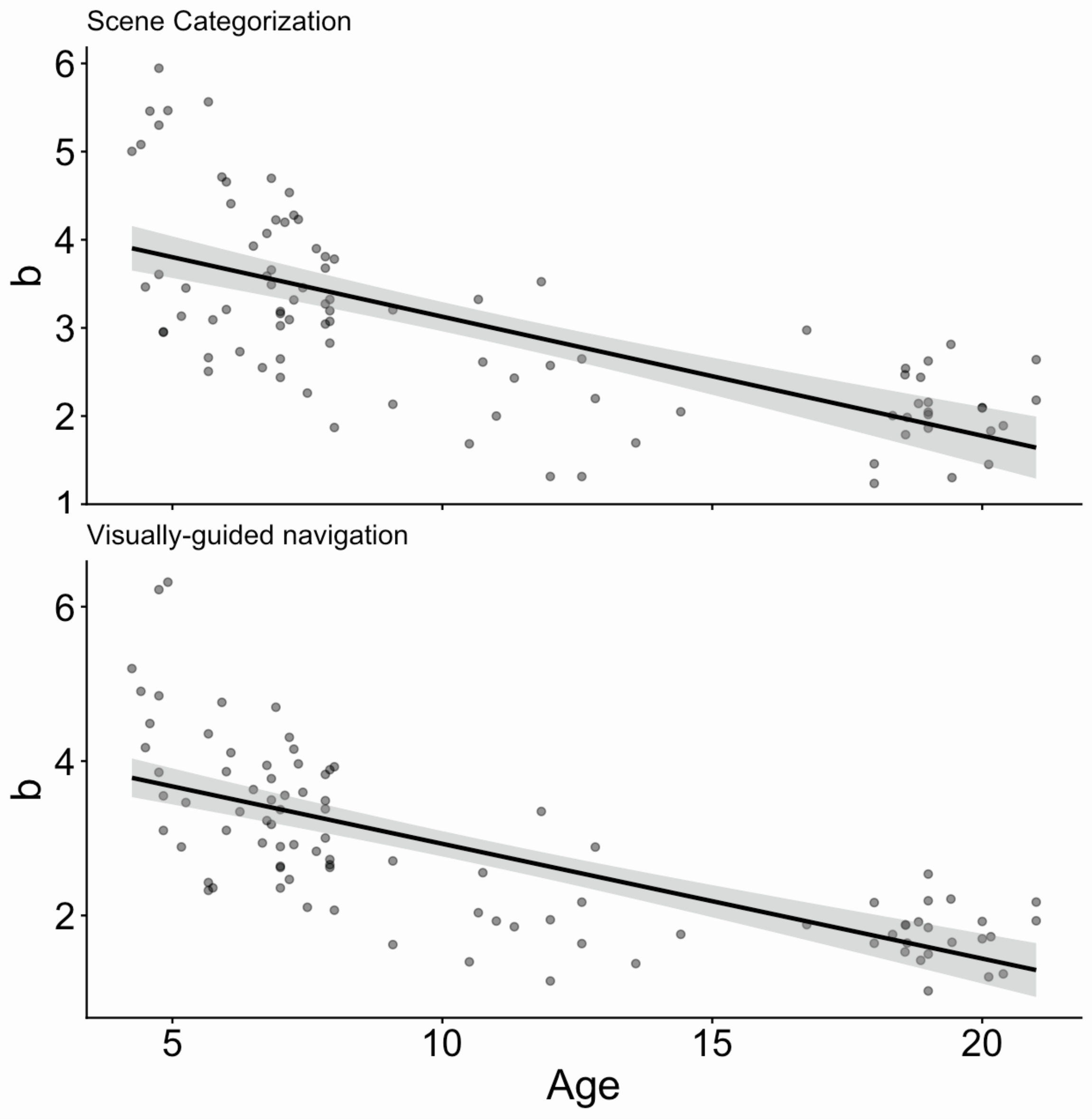

Fig. S2. Age related changes in t_{er} (nondecision time). The top panel depicts change in t_{er} over age in the scene categorization task and the bottom panel depicts change in t_{er} in the visually-guided navigation task. Related to Figure 4 and STAR Methods.

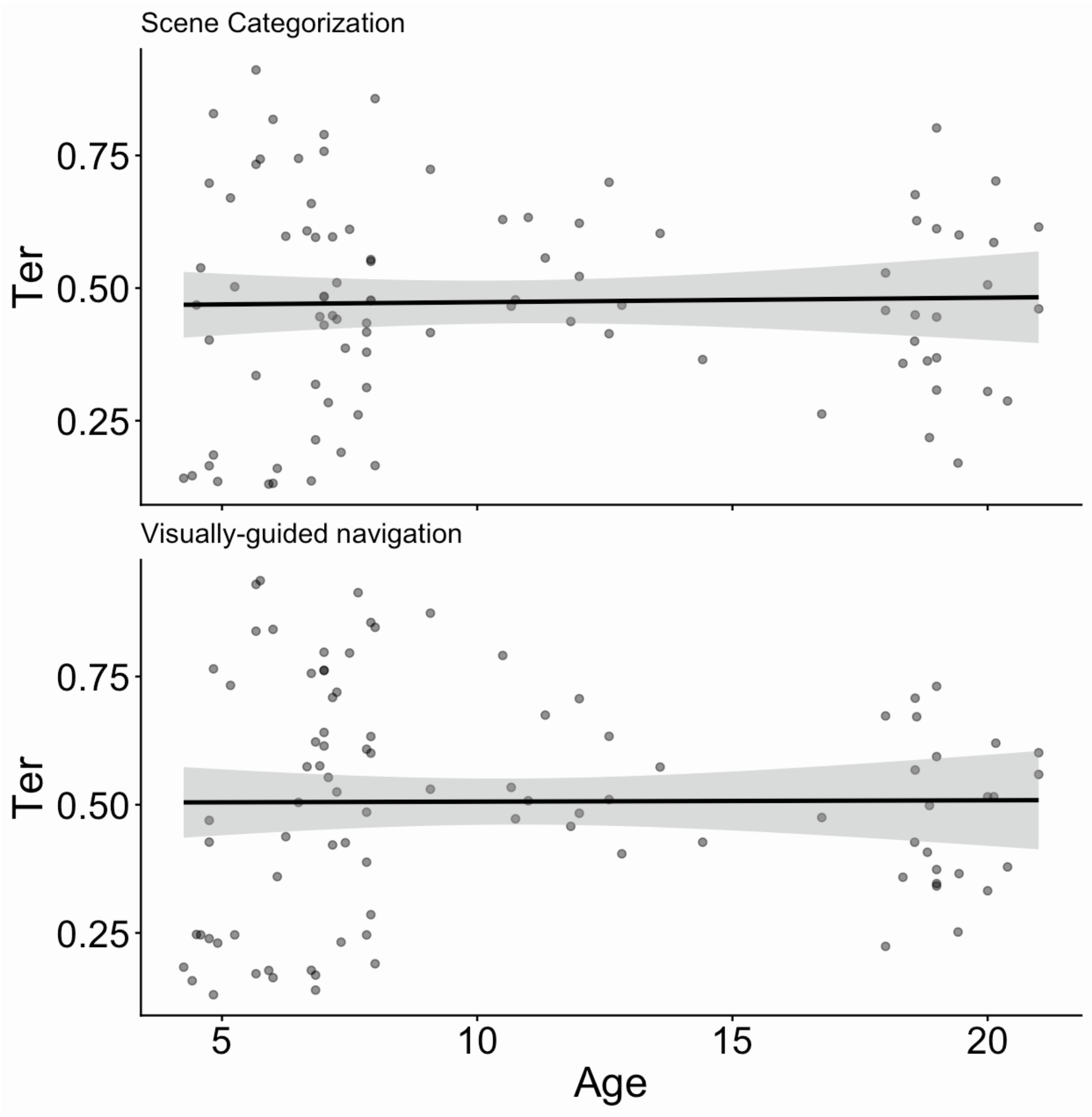

Fig. S3. Results from the parameter recovery study. Scatterplots show the relationship between model parameters estimated from empirical data and the mean recovered parameter values estimated from three simulated datasets as compared to the solid diagonal line indicating perfect recovery. Numbers above each plot report the correlation coefficient (r) between the original model parameter and the mean recovered parameter. Related to STAR Methods.

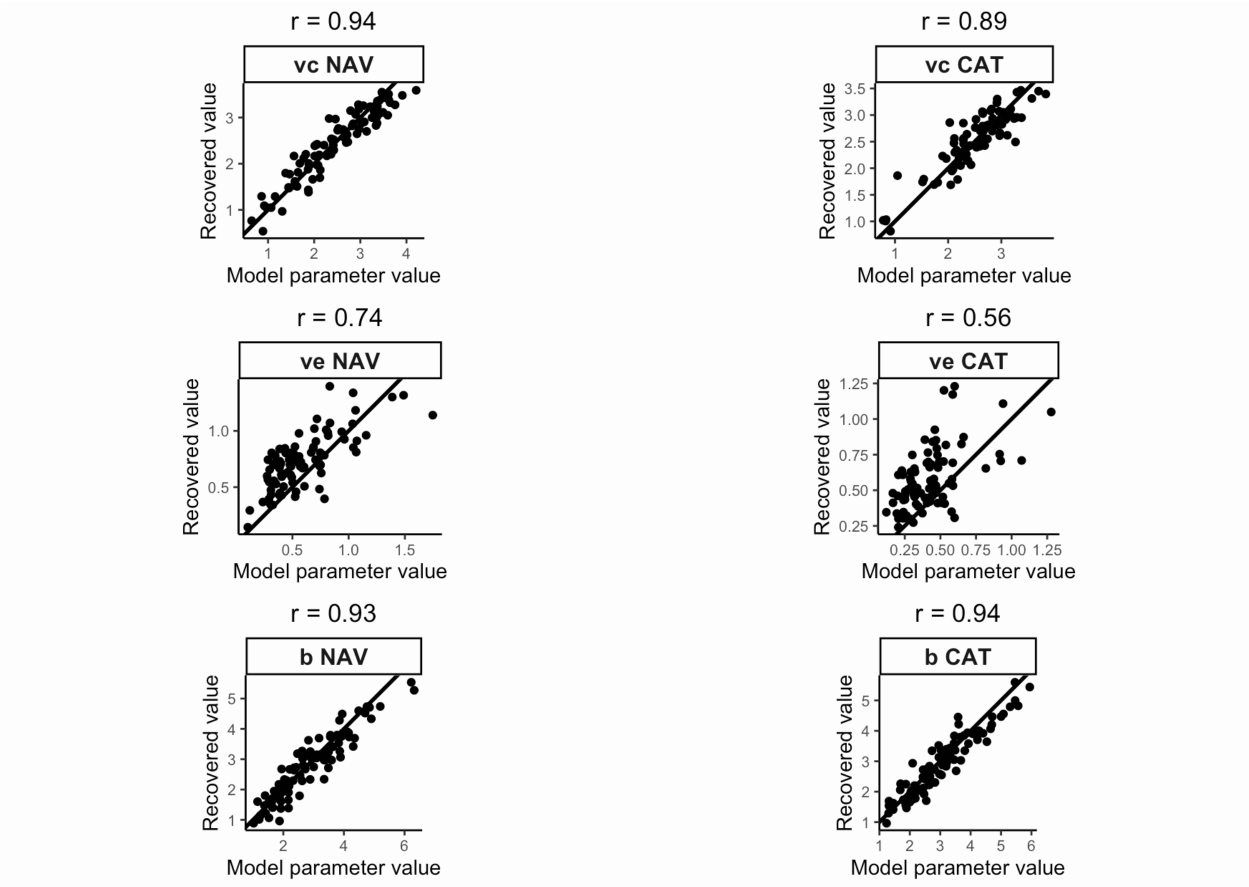

Tables

Table S1. Mean parameter values across tasks.

Parameter	Task	Mean sample value
vc	Navigation	2.53
	Categorization	2.52
ve	Navigation	.42
	Categorization	.58
b	Navigation	2.84
	Categorization	3.06
sd vc	-	1.00
sd ve	-	.75
t0	Navigation	.46
	Categorization	.50
st0	-	.32

Data S1/Methods S1: Supplemental Methods

Modeling approach. Samples for vc and ve were estimated separately for each condition ("easy" and "hard") for each participant and each task. We also calculated the LBA threshold, B, for each response option within a task (e.g., bedroom, kitchen and living room in the scene categorization task, and left, right, center in the visually-guided navigation task). In addition to the above parameters, we also estimated additional parameters as is typical in modeling the LBA, including the upper threshold of the start point (A), variability in correct drift rate (sd vc), variability in error drift rate (sd ve), nondecision time (t0), and variability in nondecision time (st0). Next, sd vc was fixed to 1 to scale the remaining parameters.

Next, we computed the mean vc and ve sample values across the difficulty conditions in each task. A was added to B to create boundary parameters (b) comparable those used in other evidence accumulation models (e.g., DDM). We then calculated a mean b for the scene categorization task and a mean b for the visually-guided navigation tasks. Finally, following previous work with the LBA(1, 2), relatively broad and uninformative priors were posited for both tasks. Mean parameter values are presented in Table S1.

Parameter Recovery. For each participant, we used the individual parameter values obtained here ("empirical parameters") to generate three sets of simulated data, each matching the number of trials each participant completed on the task. These simulated data were then used to obtain three new sets of individual level parameter values ("recovered parameters") for each participant, averaged across the sets. Next, these mean recovered parameter estimates were compared with the empirical parameter values. Associations between the recovered and empirical parameter values are illustrated in Figure S1, along with the corresponding r value for each association. For each task, parameter values showed acceptable-good recovery (all values falling between .56-.94, with the majority falling within the good recovery range). Moreover, inspection of the scatterplots in Figure S1 reveal no apparent biases in recovery. Thus, we are able to reliably recover LBA model parameters from our experimental paradigm.

SI References

1. B. M. Turner, P. B. Sederberg, S. D. Brown, M. Steyvers, A method for efficiently sampling from distributions with correlated dimensions. *Psychological methods* **18**, 368 (2013).
2. A. Weigard, C. Huang-Pollock, A. Heathcote, L. Hawk, N. J. Schlienz, A cognitive model-based approach to testing mechanistic explanations for neuropsychological decrements during tobacco abstinence. *Psychopharmacology* **235**, 3115–3124 (2018).
